# Influence of Exposure to Imidacloprid on Survivorship, Reproduction and Vitellin Content of the Carmine Spider Mite, *Tetranychus cinnabarinus*

**DOI:** 10.1673/031.010.2001

**Published:** 2010-03-16

**Authors:** Chun-Xiang Zeng, Jin-Jun Wang

**Affiliations:** Key Laboratory of Entomology and Pest Control Engineering, College of Plant Protection, Southwest University, Chongqing 400716, P.R. China

**Keywords:** neonicotinoid insecticide, sublethal effect, pest mite, *Vigna unguiculata*

## Abstract

Occasional reports linking neonicotinoid insecticide applications to field population outbreaks of the spider mite have been a topic of concern for integrated pest management programs. To elucidate the impacts of a neonicotinoid insecticide on the carmine spider mite, *Tetranychus cinnabarinus* Boisduval (Acari: Tetranychidae), the survivorship, reproduction, and vitellin contents of the mite were investigated after exposure to various concentrations of imidacloprid on the *V. unguiculata* leaf discs at 25°C, 80% RH and a photoperiod of 14:10 (L:D) in the laboratory. The results showed that the field-relevant dose of imidacloprid did not significantly affect the hatch rate of eggs or pre-imaginal survivorship of the mite, while sublethal doses of imidacloprid, previously determined for *Myzus persicae*, led to a significant increase in the hatch rate of eggs and pre-imaginal survivorship of the mite compared to the untreated control. Adult longevity and fecundity of *T. cinnabarinus* for imidacloprid-treated populations were slightly prolonged and increased, respectively, but the difference from the untreated control was not significant. The vitellin content in eggs increased significantly after exposure to imidacloprid. Imidacloprid may be one of the major reasons for the outbreak of *T. cinnabarinus* in the field.

## Introduction

Neonicotinoid insecticides were introduced into the market in the early 1990s and, today, are one of the most important chemical groups used to control sucking insects as the major insecticide replacing organophosphate and carbamate insecticides. Imidacloprid, the first neonicotinoid insecticide, is particularly effective against sucking insects such as aphids and whiteflies, as well as several beetles, flies, and moth species, with its systemic and broad-spectrum activities; imidacloprid, however, is not toxic to phytophagous mites at normal field rates (Elbert et al. 1991, Nauen et al. 2001, Nauen and Bretschneider 2002).

Imidacloprid has a mixed reputation regarding its safety to natural enemies of pests (James and Price 2002). It was reported that using imidacloprid against sucking insects is safe for natural enemies of other pests such as spiders and some predatory beetles and bugs (Hough-Goldstein and Whalen 1993, James 1997, Kunkel et al. 1999; James and Vogele 2001, Elzen 2001), but other studies showed that imidacloprid was highly toxic to certain species of spiders, predatory beetles, and bugs (Mizell and Sconyers 1992, Stark et al. 1995, Delbeke et al. 1997, Sclar et al. 1998, James and Coyle 2001). In addition, the application of systemic insecticides, as well as some non-systemic contact poisons like DDT and several synthetic pyrethroids, in fields often causes resurgence of non-target pest insects and mites. Recently, field outbreaks of the two-spotted spider mite, *Tetranychus urticae*, following acetamiprid (Assail) treatments have been reported (Beers et al. 2005). Moreover, in laboratory experiments, James and Price (2002) reported a significant increase in egg production in *T. urticae* after spray and systemic applications of imidacloprid at field-relevant rates for hop yards. In contrast, significantly reduced oviposition in *T. urticae* following drench or foliar applications of imidacloprid and acetamiprid at field-relevant rates was reported in another study (Ako et al. 2004). Ako et al. (2006) suggested that the ovipositional response of *T. urticae* to field-recommended doses of imidacloprid is strain-dependent. It is generally understood that imidacloprid and, most likely, neonicotinoids in general, used at their field-recommended rates, are not the sole factors contributing to the propagation of mite pests by oviposition stimulation, and one possible explanation currently under investigation is interspecies competition.

The carmine spider mite, *Tetranychus cinnabarinus* Boisduval (Acari: Tetranychidae), is a polyphagous spider mite pest of vegetable crops and ornamental plants in warm zones throughout the world (He 1990). *T. cinnabarinus* is an important mite pest of horticultural and field crops in Southwestern China and is exposed to imidacloprid in many crop systems, particularly those that have aphids and whiteflies as principal pests. *T. cinnabarinus* shares the same ecological niche with several other important pests, such as aphids and whiteflies, in greenhouses and the open field. When these insects are chemically controlled, *T. cinnabarinus* is a non-target pest insect that is also exposed to imidacloprid. Currently, research on the impact of imidacloprid on *T. cinnabarinus* is lacking. Therefore, the present study was undertaken to assess the potential effects of imidacloprid on the survivorship, reproduction, and vitellin content of the carmine spider mite through a laboratory experiment.

## Materials and Methods

### Mites

The stock culture of the carmine spider mite, *T. cinnabarinus*, was collected from the cowpea bean, *Vigna unguiculata* Endlicher (Fabales: Fabaceae), in 2003 in Chongqing, China. This culture was maintained on potted *V. unguiculata* plants in a walk-in insect rearing room at 28 ± 1°C, 75–80% relative humidity, and a photoperiod of 14:10 hours (L:D). This colony was maintained for more than two years without the use of any pesticide. In order to obtain homogeneous individuals for the experiments, a synchronized mite culture was established in 2006 on *V. unguiculata* in the greenhouse with the same conditions as insect rearing room. Thirty mated female mites were placed on the third leaf from the top of *V. unguiculata* plants (three females each) on the 12 h. Thereafter, the adults were removed, and the offspring were kept until the progeny had developed into preovipositional females. Cotton strips were used to keep the mites from escaping. In order to increase the number of male mites and thereby increase the mating chance for young pre-ovipositional females, 15 additional males collected from the stock culture were transferred to the *V. unguiculata* mite rearing plants before the deuteronymphal stage of the synchronized population.

### Bioassays

Imidacloprid (10% WP, Jiangsu Wujiang Pesticide Ltd. Co., China) was tested at sublethal doses (0.5778, 1.4247, and 2.7308 mg/L, corresponding to LD_10_, LD_20_, and LD_30_ respectively and the field-relevant dose (23.4730 mg/L) previously determined for *Myzus persicae* (Zeng and Wang 2007). Approximately 100–150 young mated female mites were transferred to 10 *V. unguiculata* leaf discs (diameter 1 cm) that were placed on a moistened sponge in the Petri dish (diameter 15cm) for 24 h. The adults were then removed, and each leaf disc with 30 eggs was immersed in treatment concentrations of imidacloprid for 10 s. Tap water immersion was used as the control. The hatch rate of the carmine spider mite was observed daily for 10 d. A total of 250 hatched nymphs for each treatment was kept under aforementioned conditions until they developed into pre-ovipositional females. Pre-imaginal survivorship was determined by recording the number of offspring that survived until adulthood in each treatment. Then, 100 female mites from each treatment with the cotton strip were transferred individually to *V. unguiculata* leaf discs as previously described. Males also were introduced to each female for mating. The number of eggs oviposited was counted under a stereomicroscope using a manual counter every 2 d for 16 d. Female mites that escaped or died were excluded from the analysis. After recording the number of eggs, the female mite was transferred carefully with a soft brush from the previous leaf disc to a new leaf disc. Each treatment was replicated three times temporally.

### Determination of vitellin content

Three hundred eggs from the above treatment were homogenized manually in 200 µ l NaCl solution (0.4 M) and centrifuged at 10,000 g for 15 min at 4°C. The resulting supernatants were used to determine the content of vitellin of the carmine spider mite using bovine serum albumin as a standard (Abdel-Aal et al. 1990). Absorbance was read in the spectrophotometer with the wavelength of 595 nm. The determination also was replicated three times as a bioassay.

### Statistical analyses

Difference in hatch rate of eggs, preimaginal survivorship, fecundity, and vitellin content of the carmine spider mite were subjected to analysis of variances (ANOVA) by using the SPSS 10.0 for Windows (SPSS 1999). General linear model procedure was used and means were separated by Fisher protected least significant difference (LSD) test when significant *F*-values were obtained (p < 0.05). The percentage of the egg hatch rate was transformed to the arcsin squareroot before analysis to stabilize error variance.

## Results

### Hatch rate of eggs and pre-imaginal survivorship

**Figure 1.**
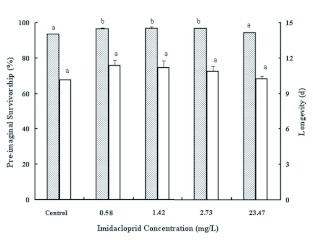
Effects of continual exposure to imidacloprid on the pre-imaginal survivorship (shaded columns) and adult longevity (empty columns) of *Tetranychus cinnabarinus* to adult. Columns marked with the same letter do not differ significantly (p < 0.05; Fisher LSD test). High quality figures are available online.

Compared with the control, exposure to the field-relevant dose of imidacloprid (23.47 mg/L) did not significantly affect the hatch rate of *T. cinnabarinus* eggs. However, exposure to the sublethal dose rates (0.5778, 1.42, and 2.73 mg/L) significantly increased the age-specific egg hatch rate (Table 1). The highest total hatch rate (95.23%) was observed with exposure to 1.42 mg/L imidacloprid. In general, the pre-imaginal survivorship was relatively high in all treatments. Compared with the control, the pre-imaginal survivorship was significantly higher with the exposure to the three sublethal doses of imidacloprid; however, no differences in pre-imaginal survivorship were observed between the control and the field-relevant dose of imidacloprid (*F* =24.64; df = 4, 10; p < 0.001; Figure 1).

### Adult fecundity and longevity

The age-specific adult fecundity and longevity of *T. cinnabarinus* under the impact of imidacloprid is presented in Table 2 and Figure 1. In general, the observed ovipositional pattern showed an increase in egg number from the second day after adulthood (day 2) onward, with a maximum number of eggs laid at day 8 and followed by a constant decrease until the death of the female (Table 2). Compared with the control, the imidacloprid-treated populations led to a small increase in mite fecundity from day 2 to day 16, but the difference was not significant (Table 2). In most cases, the highest fecundity was observed for the 0.5778 mg/L imidacloprid-treated population. The adult longevities did not differ significantly among the treatments (*F* = 0.398; df = 4, 391; ns; Figure 1).

**Table 1.**
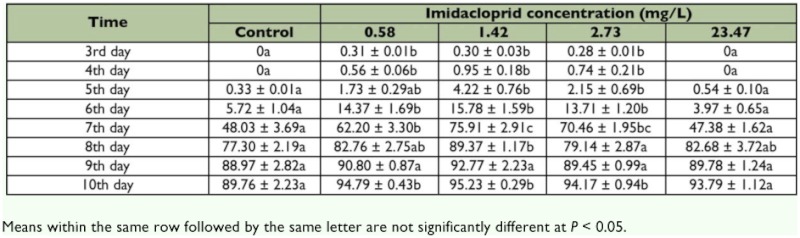
Effects of continual exposure to imidacloprid on the cumulative hatch rate of *T. cinnabarinus* eggs (Mean percentage ± SE)

### Vitellin content

**Figure 2.**
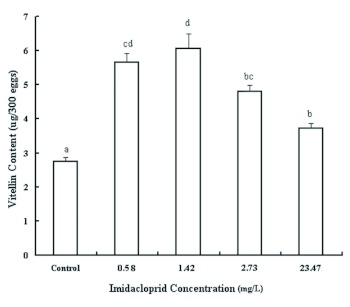
Effects of continual exposure to imidacloprid on vitellin content of *Tetranychus cinnabarinus*. Columns marked with the same letter do not differ significantly (p < 0.05: Fisher LSD test). High quality figures are available.

Compared with the control population, the vitellin contents in the imidacloprid-treated eggs were all significantly increased (*F* = 40.70; df = 4, 10; p < 0.001; Figure 2). Among the tested concentrations of imidacloprid, the highest vitellin content of *T. cinnabarinus* (6.07 µ g/300 eggs) was recorded for the 1.42 mg/L imidaclopridtreated population, and the lowest vitellin content (3.72 µg/300 eggs) was recorded for the 23.47 mg/L treated population (Figure 2).

**Table 2.**
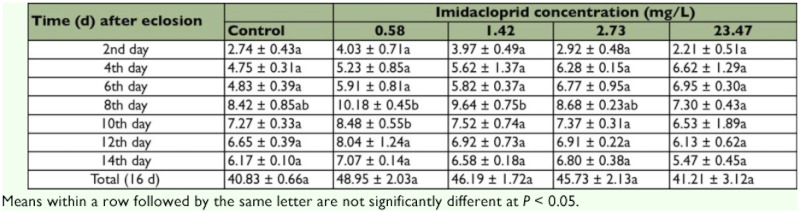
Effects of exposure to imidacloprid on the age-specific fecundity of *T. cinnabarinus* females (Mean number of eggs ± SE)

## Discussion

Previous investigation showed that there is no acute, lethal effect of imidacloprid against *T. cinnabarinus*. The mite is particularly serious in vegetable crops with aphids and whiteflies as principal pests that are controlled routinely by imidacloprid (Wang et al. unpublished data). In this study, the hatch rate of eggs, pre-imaginal survivorship, age-specific fecundity, longevity, and the vitellin content of *T. cinnabarinus* on *V. unguiculata* leaves were compared under laboratory controlled conditions to determine the impact of imidacloprid. The results showed that sublethal imidacloprid doses led to significantly earlier hatch time, greater total hatch rate, and increased pre-imaginal survivorship to adult. However, when treated with the field-relevant rate of imidacloprid, these parameters did not differ significantly from the control. Results of this study also showed that exposure to imidacloprid significantly increased the vitellin content of *T. cinnabarinus*, which, in turn, led to an increase in the speed of egg hatch and total hatch rates. The highest vitellin content was recorded at the sublethal imidacloprid dose 1.42 mg/L. The speed of hatching was also the fastest, and the total hatch rate was the highest for this population. It is known that the sublethal doses of many insecticides stimulate pest resurgence, and the cause of pest resurgence is usually due to the suppression of a natural enemy or the reproductive stimulation of pests (Morse and Zareh 1991, Nandihalli et al. 1992, Nemoto 1993). In southwestern China, imidacloprid is typically applied on commercial vegetable crop fields as foliar spraying, and the active ingredient of this chemical is systemic. The concentrations on the plants would decrease gradually to sublethal doses as the plant aged. Thus, *T. cinnabarinus* often is exposed to field-relevant or sublethal doses of imidacloprid. The present study suggests that through the stimulation of egg hatching and enhancement of the pre-imaginal survivorship, the application of imidacloprid may be one of the reasons for field outbreaks of *T. cinnabarinus* in southwestern China. However, other factors such as climate, agronomic practices, and pest resistance could also interact with imidacloprid, yielding less predictable results than those obtained under controlled conditions. Therefore, further investigation is needed, particularly under field conditions, in order to shed light on the factors which may interact with imidacloprid to lead to mite population increase.

Ako et al. (2006) reported that the fecundity of two strains of *T. urticae* (namely GSS, an acaricide-susceptible strain, and WI, an organophosphate-selected strain) treated with the field-relevant doses of imidacloprid decreased, while two other strains (namely USA, a largely uncharacterized strain, and Akita, a mitochondrial electron transport inhibitor, acaricide-resistant and cross-resistant to dicofol strain) did not differ from the untreated control. The same phenomena also were observed for sex ratio, hatch rate of eggs, and pre-imaginal survivorship of *T. urticae*. However, James and Price (2002) reported a significant increase in oviposition of *T. urticae* after drench or foliar applications of imidacloprid at concentrations of 0.011 and 0.013% A.I. in laboratory. According to Luckey (1968), the phenomenon of reproductive stimulation of pests or beneficials after exposure to sublethal doses of systemic insecticides was the basic hypothesis of hormoligosis. However, Cohen (2006) suggested that hormoligosis cannot be claimed for cases in which the observed stimulatory effects were due to exposure of non-target pests (i.e., mites) to pesticides (DDT, carbaryl, insecticidal pyrethroids or imidacloprid). Instead, pesticide-induced homeostatic modulation is suggested as a broader term to include both hormesis and stimulatory effects of pesticides on non-target pests. A possible reason for these contradicting results are differences in terms of methodology and the mites used in studies. Only one population of *T. cinnabarinus* was used in the present study, and the different reactions of the mites may be due to differences in laboratory and field studies. The present study, nonetheless, provided some basic information on the impact of imidacloprid on the survivorship, reproduction, and vitellin content of *T. cinnabarinus*. The widespread stimulation of fecundity in *T. cinnabarinus* and *T. urticae* by imidacloprid and other chloronicotinyls, if confirmed, would have great significance and importance to many crop protection and integrated pest management programs throughout the world. Since imidacloprid has a mixed reputation regarding its safety for natural enemies of pests and non-target pest species. Further, detailed studies of the impact of imidacloprid on *T. cinnabarinus* and its natural enemies will be necessary for the development of integrated pest management programs for mite control.
